# Spatial Transcriptomic Profiling of Tetraspanins in Stage 4 Colon Cancer from Primary Tumor and Liver Metastasis

**DOI:** 10.3390/life14010126

**Published:** 2024-01-15

**Authors:** Thanawat Suwatthanarak, Kullanist Thanormjit, Tharathorn Suwatthanarak, Onchira Acharayothin, Asada Methasate, Vitoon Chinswangwatanakul, Pariyada Tanjak

**Affiliations:** 1 Siriraj Cancer Center, Faculty of Medicine Siriraj Hospital, Mahidol University, Bangkok 10700, Thailand; thanawat.suw@mahidol.edu (T.S.); kullanist.tha@mahidol.ac.th (K.T.); vitoon.chi@mahidol.ac.th (V.C.); 2 Department of Surgery, Faculty of Medicine Siriraj Hospital, Mahidol University, Bangkok 10700, Thailand; tharathorn.tha@mahidol.edu (T.S.); onchira.ach@mahidol.ac.th (O.A.); asada.met@mahidol.ac.th (A.M.)

**Keywords:** spatial, transcriptomics, tetraspanins, colon cancer, stage 4

## Abstract

Stage 4 colon cancer (CC) presents a significant global health challenge due to its poor prognosis and limited treatment options. Tetraspanins, the transmembrane proteins involved in crucial cancer processes, have recently gained attention as diagnostic markers and therapeutic targets. However, their spatial expression and potential roles in stage 4 CC tissues remain unknown. Using the GeoMx digital spatial profiler, we profiled all 33 human tetraspanin genes in 48 areas within stage 4 CC tissues, segmented into immune, fibroblast, and tumor compartments. Our results unveiled diverse gene expression patterns across different primary tumor sub-regions. CD53 exhibited distinct overexpression in the immune compartment, hinting at a potential role in immune modulation. TSPAN9 was specifically overexpressed in the fibroblast compartment, suggesting involvement in tumor invasion and metastasis. CD9, CD151, TSPAN1, TSPAN3, TSPAN8, and TSPAN13 displayed specific overexpression in the tumor compartment, indicating potential roles in tumor growth. Furthermore, our differential analysis revealed significant spatial changes in tetraspanin expression between patient-matched stage 4 primary CC and metastatic liver tissues. These findings provide spatially resolved insights into the expression and potential roles of tetraspanins in stage 4 CC progression, proposing their utility as diagnostic markers and therapeutic targets. Understanding this landscape is beneficial for tailoring therapeutic strategies to specific sub-tumor regions in the context of stage 4 CC and liver metastasis.

## 1. Introduction

Colon cancer (CC) is a major global health concern and one of the leading causes of cancer-related deaths worldwide [1]. Despite advances in diagnosis and treatment, stage 4 CC remains a major challenge due to the advanced state of the disease and limited therapeutic options [2]. Understanding the molecular landscape of stage 4 CC is crucial for identifying new biomarkers and therapeutic targets, which can improve patient outcomes [3].

Tetraspanins are a family of transmembrane proteins with a similar structure, which have been known to play important roles in various cellular processes, including cell adhesion, migration, invasion, and signaling (Scheme S1, Supplementary File S1) [4]. In the context of cancer, tetraspanins have been implicated in tumor progression, metastasis, and resistance to therapy [5,6]. By forming protein complexes with various cell surface molecules, such as integrins and receptors, tetraspanins participate in the progression and metastasis of various cancers, including CC (Scheme S1, Supplementary File S1) [5,7,8]. In CC, various studies have shown specific expressions of some tetraspanins in CC cell lines and bulk tissues, indicating their potential as diagnostic markers and therapeutic targets [7,9,10]. Some tetraspanins, such as TSPAN8 and CD151, have been associated with advanced tumor stage, metastasis, and poor clinical outcomes [7,11,12]. Although several studies have investigated the expression and functional roles of tetraspanins in CC [7], their spatial gene expression patterns within stage 4 CC tissues and their potential clinical implications have not been comprehensively explored. Furthermore, in stage 4 CC, some tetraspanin genes have not yet been investigated [7].

Spatial transcriptomic analysis—a powerful technology, which combines spatial information and gene expression profiling—has emerged as a valuable tool for unraveling the complexity of tumor microenvironments [13,14,15]. By capturing the spatial distribution of gene expression within tissue sections, spatial transcriptomics enables the characterization of heterogeneous cellular landscapes [16]. Applying this technology to stage 4 CC tissues can provide unprecedented information on spatial expression patterns and functional roles of tetraspanins in this advanced disease state. Understanding the spatial context of tetraspanin expression in stage 4 CC is not only critical to advancing our knowledge of CC biology, but it also holds promise for the development of precision medicine strategies, including the identification of new biomarkers and therapeutic targets.

In this study, our objective was to investigate the spatial transcriptomic landscape of tetraspanins in stage 4 CC tissues. We used the GeoMx digital spatial profiler, which allowed spatial transcriptomic profiling of tetraspanins in the context of tissue architecture. By analyzing primary tumor and metastatic liver tissues obtained from stage 4 CC patients, we sought to elucidate the spatial expression patterns of tetraspanins within distinct tumor sub-regions, including tumor, immune, and fibroblast compartments. The focus on tumor, immune, and fibroblast compartments arises from the pivotal roles these cellular components play in the tumor microenvironment. Tumor regions provide insights into tetraspanins associated with cancer cell growth; immune regions shed light on their involvement in immune responses; and fibroblast regions help understand their role in interactions with the stromal environment. This analysis provides valuable insights into the potential functional implications of particular tetraspanins in stage 4 CC progression and their potential as diagnostic markers and therapeutic targets.

## 2. Materials and Methods

### 2.1. Collection and Preparation of Samples

Primary and/or metastatic liver specimens of CC were collected from seven patients who were over 18 years of age and diagnosed with stage 4 CC. All patients had moderately differentiated colorectal adenocarcinoma and underwent surgical resection or biopsy at Siriraj Hospital during the period of 2020–2021. The participants consisted of three women and four men with an average age of 64.14 years. All patients had liver-only metastasis. Seven primary tissues of stage 4 CC (five from the left colon and two from the right colon) were individually collected from all participants. Four metastatic liver tissues of stage 4 CC were individually derived from four patients who had liver metastasis and underwent surgical resection or biopsy at the metastasis site. Informed consent was obtained from all participants, and ethical approval was obtained from the Siriraj Institutional Review Board (certificates of approval no. Si 348/2019 and Si 105/2021). Formalin-fixed paraffin-embedded (FFPE) tissues were prepared, sectioned at a thickness of 5 µm, and mounted on Superfrost Plus slides (VWR International, Radnor, PA, USA) for spatial transcriptomic analysis and immunohistochemistry (IHC) staining. All samples were assessed and confirmed by a pathologist.

### 2.2. Spatial Transcriptomic Analysis

Three stage 4 primary CC tissues (one from the right colon and two from the left colon) and one patient-matched metastatic liver tissue were subjected to spatial transcriptomic analysis. The analysis was performed using the GeoMx digital spatial profiling (DSP) platform (NanoString Technologies, Inc., Seattle, WA, USA), according to the manufacturer’s user manuals (MAN-10150, MAN-10152, MAN-10153, and MAN-10154).

Briefly, the prepared FFPE tissue section slides were manually deparaffinized with CitriSolv (NanoString Technologies, Inc., Seattle, WA, USA) and rehydrated using a series of 100% ethanol, 95% ethanol, and phosphate-buffered saline (PBS) washes. The tissue sections were subjected to target retrieval using Tris-EDTA (pH 9.0) in a steamer, followed by PBS washing. RNA targets were exposed by incubating the tissue section slides with proteinase K solution (NanoString Technologies, Inc., Seattle, WA, USA) and washing the slides with PBS. Post-fixation of tissue sections was performed with serial washes of 10% neutral buffered formalin (NBF), NBF stop buffer, and PBS, respectively. 

For probe incubation/hybridization, UV-cleavable high-density oligonucleotide probes in the GeoMx Human Whole Transcriptome Atlas (NanoString Technologies, Inc., Seattle, WA, USA) designed to target specific RNA transcripts, including all tetraspanin genes of interest, were incubated on the tissue sections for 18 h. After incubation of the probes, stringent washes were performed using a mixture of 4X saline-sodium citrate (SSC) and 100% formamide to remove off-target probes. 

The tissue sections were blocked with Buffer W and then stained with a cocktail of fluorescently labeled morphology markers, including SYTO 13 nuclear stain, pan-cytokeratin (PanCK), CD45, and smooth muscle actin (SMA) (NanoString Technologies, Inc., Seattle, WA, USA).

The tissue section slides were loaded onto the GeoMx DSP platform. The slides were scanned to capture fluorescent images, which were used to select the regions of interest (ROI). ROIs were segmented into three compartments (PanCK+, CD45+, and SMA+) or areas of illumination/interest (AOI). Within the instrument, the UV-cleaved oligos from the AOIs were collected into the wells of a collection plate. 

The collected aspirates were dried, rehydrated with nuclease-free water, and transferred to a polymerase chain reaction (PCR) plate. Library preparation was performed using Seq Code primers (NanoString Technologies, Inc., Seattle, WA, USA). The products were pooled and purified using AMPure XP beads (Beckman Coulter, Inc., Brea, CA, USA). The quality and quantity of the library were evaluated using the Agilent TapeStation platform (Agilent Technologies, Santa Clara, CA, USA). The qualified library, diluted to 250 pM, was then sequenced on the NovaSeq 6000 instrument (Illumina, Inc., San Diego, CA, USA).

The obtained FASTQ sequencing files were processed into digital count conversion (DCC) files using the GeoMx next-generation sequencing (NGS) pipeline with NanoString’s standalone software (Version 2.0.0.15, NanoString Technologies, Inc., Seattle, WA, USA). The DCC files were next uploaded to the GeoMx DSP platform. Thus, quality control checks and data analyses were performed. 

For data analysis, the obtained data were normalized to the third quartile (Q3). The effectiveness of the Q3 normalization method lies in its ability to reliably reduce the influence of outliers on spatial transcriptomic data. Unlike mean-based normalization, which can be sensitive to extreme values, the Q3 method effectively normalizes expression data by utilizing the interquartile range. This approach is particularly advantageous when dealing with spatial transcriptomic datasets, which may exhibit spatially heterogeneous gene expression patterns. By focusing on the central tendency of data distribution, the Q3 normalization method ensures a more accurate representation of gene expression levels across distinct cellular regions. Therefore, Q3 normalization adjusts the gene expression data to ensure uniform gene expression ranges across all segments, minimizing variations arising from segment size, cellularity, and other technical factors. It is most suitable for a probe panel, which is large and diverse, particularly one targeting the whole transcriptome. It is the recommended normalization method for all targets above the limit of quantitation (LOQ), according to the NanoString guidelines. The process involves dividing the counts in one segment by its third quartile value, followed by multiplying that value by the geometric mean of the third quartile values of all segments. The Q3 normalized target count matrix is shown in Supplementary File S2. The expression patterns of tetraspanins within distinct tumor regions, including the tumor, immune, and fibroblast regions, were then analyzed to characterize their spatial expression landscapes.

Cellular deconvolution was performed in each AOI by applying the processed gene expression data to the Gene Expression Deconvolution Interactive Tool (GEDIT) (https://webtools.mcdb.ucla.edu/, accessed on 8 August 2023) [17]. Cellular deconvolution results are shown in Supplementary File S1.

ImageJ software (Version v.153, National Institutes of Health, Bethesda, MD, USA) was also employed for quantifying the immunofluorescence (IF) intensity of CD45, SMA, and PanCK in the representative images of primary and metastatic liver tissues of stage 4 CC.

Statistical analysis was performed using IBM SPSS Statistics (Version 18.0, SPSS Inc., Chicago, IL, USA) and/or Prism 8.0 (GraphPad Software, Inc., San Diego, CA, USA) according to data type and distribution. Some results of the statistical analysis are included in Supplementary File S1 (Tables S1 and S2). A *p*-value of <0.05 was considered statistically significant. The Kruskal–Wallis H-test was applied for spatial transcriptomic profiling of tetraspanins in stage 4 primary CC tissues. The Mann–Whitney U-test was used for analyzing spatially resolved differential gene expression levels of tetraspanins in primary and metastatic liver tissues of stage 4 CC. The *T*-Test or Mann–Whitney U-test was utilized to quantify the IF intensity of CD45, SMA, and PanCK in IF images of primary and metastatic liver tissues of stage 4 CC.

### 2.3. IHC Staining of TSPAN8 Protein in Primary and Metastatic Liver Tissues of Stage 4 CC

Seven stage 4 primary CC tissues and four metastatic liver tissues were subjected to IHC staining. A FFPE tissue section slide was manually deparaffinized using m-Xylene (Sigma-Aldrich Corp, Saint Louis, MO, USA) and rehydrated using a series of 100% ethanol, 95% ethanol, and PBS washes. The prepared FFPE slide was dried, outlined with a hydrophobic barrier around the tissue, and then covered with a blocking solution (Dako, Agilent Technologies, CA, USA). The tissue was positioned in a humidity chamber and covered with a diluted primary antibody solution (1/100, anti-TSPAN8, ab230448, Abcam, Cambridge, UK) at 4 °C overnight. Subsequently, the tissue was treated with Dako HRP solution (30 min) and then Dako DAB solution (5 min) in a light-protected humidity chamber. Finally, the tissue was exposed to a solution of Dako hematoxylin (5 min), washed with water, mounted with ProLong Gold Antifade Mountant (Thermo Fisher Scientific, Waltham, MA, USA), and subjected to scanning using the Leica Asperio ScanScope CS system (Leica Camera AG, Wetzlar, Germany). ImageJ software (Version v.153, National Institutes of Health, Bethesda, MD, USA) was employed for quantifying IHC intensity in the images obtained. The *p*-value was calculated using the Mann–Whitney U-test.

## 3. Results

### 3.1. Spatial Transcriptomic Profiling of Tetraspanins in Stage 4 Primary CC Tissues

**Figure 1:**
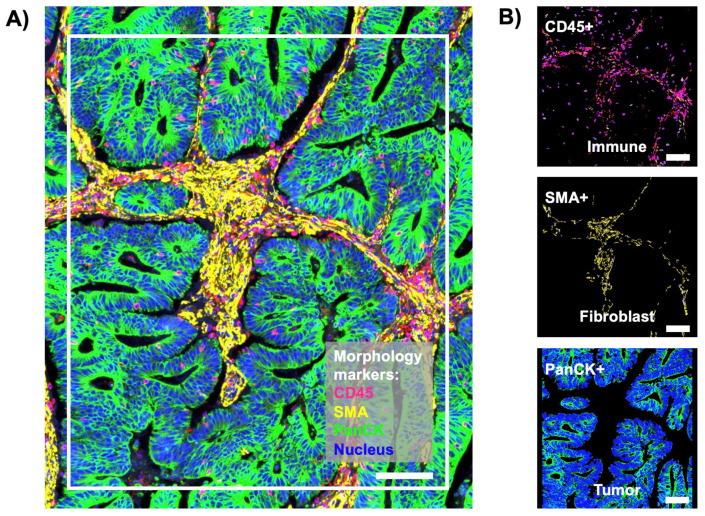
(**A**) A representative fluorescence (IF) image of the region of interest (ROI) in the primary tumor (white rectangle) from spatial transcriptomic profiling of tetraspanins in stage 4 primary CC tissues. Pink color is CD45 staining and the immune compartment. Yellow is the staining with smooth muscle actin (SMA). Green is PanCK staining and the tumor compartment. Blue is SYTO13 staining and the nucleus. (**B**) Fluorescence images of the segmented areas of interest (AOI) in (**A**), including immune (CD45+, top), fibroblast (SMA+, middle), and tumor (PanCK+, bottom) compartments. Scale bar, 100 µm.

Spatial transcriptomic profiling of tetraspanins in stage 4 primary CC tissues would provide valuable insights into the expression and distribution of these cell surface proteins within the tumor microenvironment. First, we examined the spatial transcriptomic profiling of all 33 human tetraspanins in 36 AOIs in stage 4 primary CC tissues. Figure 1A shows a representative fluorescence image of ROI in the primary tumor. Each ROI was segmented into three AOIs for CD45+, SMA+, and PanCK+ (Figure 1B). CD45—also known as leukocyte common antigen—is a cell surface protein found in all nucleated hematopoietic cells, including immune cells, such as lymphocytes, monocytes, and granulocytes [18]. Therefore, the CD45 stain (pink) identifies the immune compartment in tissues (Figure 1A,B). SMA staining (yellow) is often indicative of the presence of SMA protein and smooth muscle cells or myofibroblasts [19], indicating the fibroblast compartment (Figure 1A,B). Staining PanCK or Pan-Cytokeratin (green), which is a group of proteins found in the cytoskeleton of epithelial cells, identifies epithelial tumor cells within tissue samples (Figure 1A,B) [20]. As a result of human whole-transcriptome-based cell deconvolution, the computational results showed that the tumor compartment (PanCK+) mainly contained colorectal adenocarcinoma (Figure S1, Supplementary File S1). Macrophages, mast cells, CD4+ alpha beta T cells were the main populations in the immune compartment (CD45+) (Figure S1, Supplementary File S1). Furthermore, the fibroblast compartment (SMA +) consisted abundantly of fibroblasts (Figure S1, Supplementary File S1). These cell deconvolution results indicated the successful segmentation and cell-type compositions in the segmented compartments.

**Figure 2:**
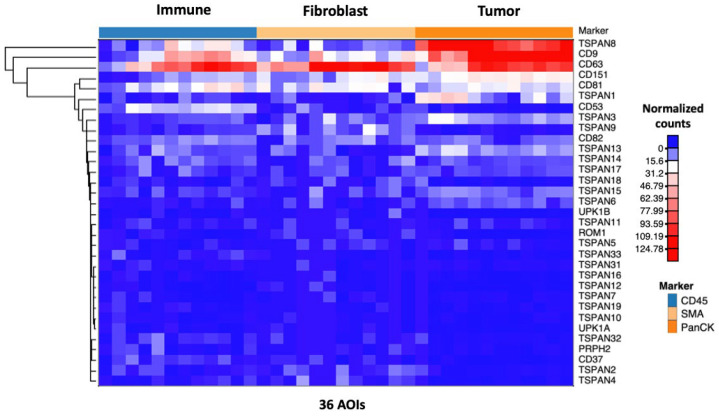
The heatmap illustrates the Q3 normalized gene counts of all tetraspanin targets across the 36 areas of interest (AOIs) within the primary tumors. The heatmap was generated using NG-CHM BUILDER, accessible at https://build.ngchm.net/NGCHM-web-builder/ (accessed on 6 September 2023). CD45 represents the immune compartment; SMA corresponds to the fibroblast compartment; and PanCK designates the tumor compartment.

To study the spatial transcriptomic profile of tetraspanins in tissues of primary stage 4 CC, the expression of all tetraspanin genes was analyzed in each segmented AOI. Figure 2 illustrates the normalized expression level of all 33 tetraspanin genes in all 36 AOIs. The spatial transcriptomic result revealed distinct expression patterns of tetraspanins within different sub-tumor regions of stage 4 primary CC tissues (Figure 2). Particular tetraspanin genes were specifically overexpressed in each sub-tumor region, suggesting the spatial heterogeneity of tetraspanins in the primary tumor (Figure 2). 

**Figure 3:**
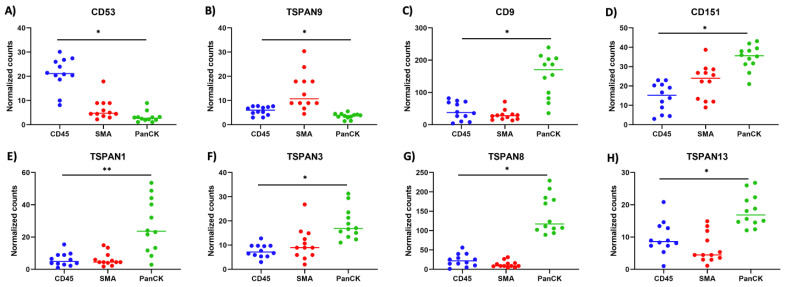
The plots depict the differential gene expression levels of the top 25% tetraspanin expressers in each region, comprising (**A**) immune (CD45), (**B**) fibroblast (SMA), and (**C**–**H**) tumor (PanCK) compartments, within the primary tumors. The *p*-value was calculated using the Kruskal–Wallis H-test. *, *p*-value < 0.001, and **, *p*-value < 0.01.

To further study the spatial heterogeneity of tetraspanin genes in the primary tumor, the top 25% of tetraspanin expressers in each compartment were focused and compared with the other two compartments. Figure 3 shows differentially expressed levels of the top 25% of tetraspanin expressers in each region, including the immune (Figure 3A), fibroblast (Figure 3B), and tumor compartments (Figure 3C–H). In the tumor compartment, several tetraspanins, including CD9, CD151, TSPAN1, TSPAN3, TSPAN8, and TSPAN13, exhibited specific overexpression levels, indicating their potential involvement in tumor growth and proliferation (Figure 3C–H). In the fibroblast compartment, TSPAN9 was significantly overexpressed, suggesting its potential role in tumor invasion and metastasis (Figure 3B). In the immune compartment, CD53 was specifically overexpressed, suggesting its involvement in immune modulation (Figure 3A). When comparing left-sided and right-sided stage 4 primary CC tissues, TSPAN1 and CD9 were identified as differentially expressed genes among those with overexpression, as depicted in Figure S2 (Supplementary File S1). Due to the anatomical and physiological differences, TSPAN1 and CD9 may play distinct roles or have differential regulatory mechanisms in the context of left-sided and right-sided stage 4 primary CC. This spatial heterogeneity suggests that tetraspanins have diverse roles in different aspects of CC progression, including proliferation, invasion, and interactions with the tumor microenvironment. These results demonstrate that these overexpressed tetraspanins had the potential to be involved and could be used as promising biomarkers and therapeutic targets for related sub-tumor regions in stage 4 primary CC. This finding also underscores the complex nature of CC and the need for a spatially resolved approach to comprehensively understand the molecular landscape.

### 3.2. Spatially Resolved Differential Gene Expression Levels of Tetraspanins in Primary and Metastatic Liver Tissues of Stage 4 CC

**Figure 4:**
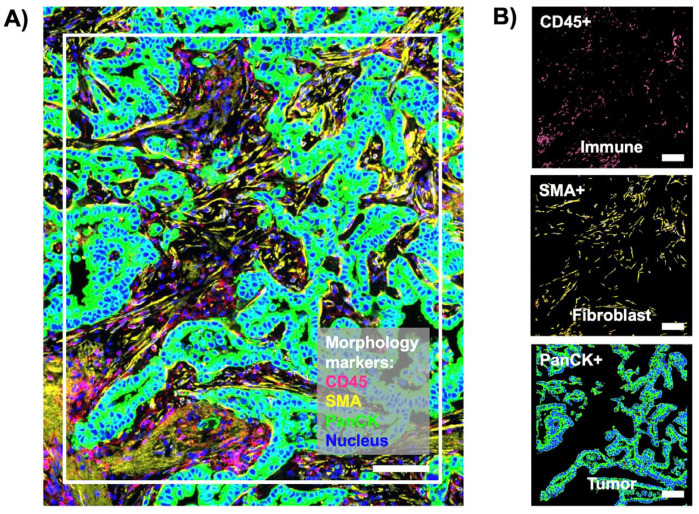
(**A**) A representative fluorescence (IF) image of the region of interest (ROI) in metastatic liver tumor (white rectangle) from spatial transcriptomic profiling of tetraspanins in metastatic liver tumor of stage 4 CC. Pink color is CD45 staining and the immune compartment. Yellow is the staining with smooth muscle actin (SMA). Green is PanCK staining and the tumor compartment. Blue is SYTO13 staining and the nucleus. (**B**) Fluorescence images of the segmented areas of interest (AOI) in (**A**), including immune (CD45+, top), fibroblast (SMA+, middle), and tumor (PanCK+, bottom) compartments. Scale bar, 100 µm.

The spatial transcriptomic analysis of tetraspanins in primary CC and metastatic liver tumors would provide valuable insights into the spatial distribution and alterations in these cell surface proteins within the tumor microenvironment. This would be useful for understanding the role of tetraspanins in CC progression, metastasis, and interactions with the liver microenvironment. Therefore, we identified specific tetraspanin genes, which exhibited spatially resolved differential expression patterns between patient-matched primary and metastatic liver tissues of stage 4 CC. Figure 4A shows a representative fluorescence image of ROI in metastatic liver tumor. Each ROI was also segmented into three AOIs for CD45+ (immune compartment), SMA+ (fibroblast compartment), and PanCK+ (tumor compartment) (Figure 4B). According to the IF images, the quantified IF intensity of CD45, SMA, and PanCK showed no significant difference between primary and metastatic liver tissues of stage 4 CC (Figure S3, Supplementary File S1). Based on cell deconvolution results, colorectal adenocarcinoma was the main population still in the tumor compartment of metastatic tumor, confirming the presence of CC in metastatic liver tumor (Figure S4, Supplementary File S1). Similarly, fibroblasts were abundantly found in the fibroblast compartment of metastatic liver tumor (Figure S4, Supplementary File S1). However, the population of CD4+ alpha beta T cells increased in the immune compartment of metastatic tumor compared to the primary tumor (Figure S4, Supplementary File S1). Furthermore, the macrophage population M1 decreased in the immune compartment of metastatic tumor compared to the primary tumor (Figure S4, Supplementary File S1). This suggests that key changes in cell sub-populations were found in the immune compartment of metastatic tumor compared to the primary tumor.

**Figure 5:**
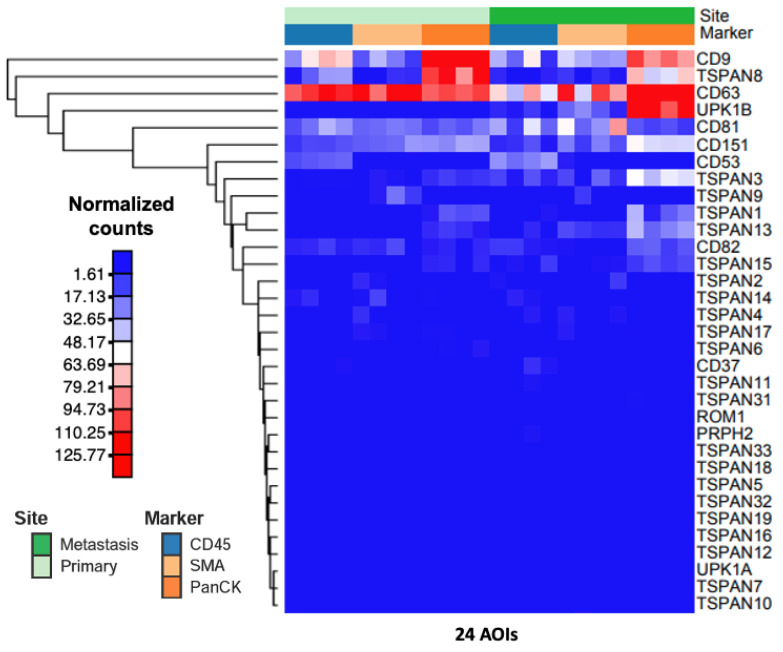
The heatmap displays the Q3 normalized gene counts of all tetraspanin targets across the 24 areas of interest (AOIs) within both primary and matched metastatic liver tumors. The heatmap was generated using NG-CHM BUILDER, accessible at https://build.ngchm.net/NGCHM-web-builder/ (accessed on 6 September 2023). CD45 represents the immune compartment; SMA corresponds to the fibroblast compartment; and PanCK designates the tumor compartment.

**Figure 6:**
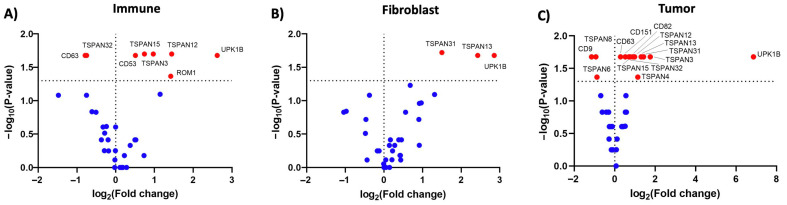
The volcano plots illustrate the differentially expressed levels of tetraspanin genes in (**A**) immune, (**B**) fibroblast, and (**C**) tumor compartments of the metastatic liver lesion compared to its primary CC tumor. The *p*-value was calculated using the Mann–Whitney U-test.

To compare the spatial transcriptomic profiles of tetraspanins in primary and metastatic liver tissues of stage 4 CC, the expression of all tetraspanin genes was analyzed in each segmented AOI of matched primary and metastatic tissues. Figure 5 shows the normalized counts of all tetraspanin genes in 12 AOIs in the primary tumor and 12 AOIs in the metastatic liver tumor. As shown in Figure 5, the heatmap revealed significant alterations in the expression levels of tetraspanin genes in stage 4 primary CC compared with liver metastasis. Figure 6 shows the volcano plots of differentially expressed levels of all tetraspanins in all sub-tumor regions, including the immune, fibroblast, and tumor compartments. Genes CD53, ROM1, TSPAN3, TSPAN12, TSPAN15, and UPK1B exhibited significantly upregulated expression in the immune compartment of metastatic tumor (Figure 6A). The upregulation of these tetraspanins in immune cells within the metastatic tumor suggests that they may influence immune cell activation and responses against the tumor. Conversely, CD63 and TSPAN32 were downregulated in the immune compartment of metastatic tumor (Figure 6A). Downregulation of CD63 and TSPAN32 in immune cells within the metastatic tumor may be related to reduced immune cell activation and responsiveness. The upregulation and downregulation of these tetraspanin genes may result from the difference in immune cell population between the primary and metastatic tumors, as described above. This finding in the metastatic liver tumor provides information on the distribution of immune cells expressing tetraspanin within liver metastasis, which differed from the immune compartment of primary tumor. The alterations in tetraspanin in immune cells may be beneficial to understanding how tetraspanins influence the immune responses and immune cell infiltration in primary and metastatic tumors.

The TSPAN13, TSPAN31, and UPK1B genes were upregulated in the fibroblast compartment of metastatic tumor (Figure 6B). The upregulated expression alterations in these tetraspanins in the fibroblasts in metastatic tumor may be associated with their relevance to tumor–fibroblast interactions, metastasis, and unique features of the metastatic niche. The tetraspanin gene was not downregulated in the fibroblast compartment of metastatic tumor (Figure 6B).

Several tetraspanin genes, including CD63, CD82, CD151, TSPAN3, TSPAN4, TSPAN12, TSPAN13, TSPAN15, TSPAN31, TSPAN32, and UPK1B, showed significantly upregulated expressions in the tumor compartment of the metastatic site (Figure 6C). These tetraspanins, upregulated in tumor cells within metastatic tumor, may be associated with the enhanced metastatic potential and liver-specific metastatic processes. On the other hand, CD9, TSPAN6, and TSPAN8 were downregulated in the tumor compartment of metastatic tumor (Figure 6C). These tetraspanins, downregulated in tumor cells within metastatic tumor, may be related to tumor cell behavior within the liver microenvironment.

These results show alterations in the spatial transcriptomic landscape of tetraspanins between patient-matched primary and metastatic liver tissues of stage 4 CC. Spatially resolved alterations in tetraspanin expression in primary and metastatic liver tissues of stage 4 CC not only show heterogeneity but can also lead to the discovery of novel therapeutic targets and strategies for inhibiting metastasis.

### 3.3. IHC Staining of TSPAN8 Protein in Primary and Metastatic Liver Tissues of Stage 4 CC

**Figure 7:**
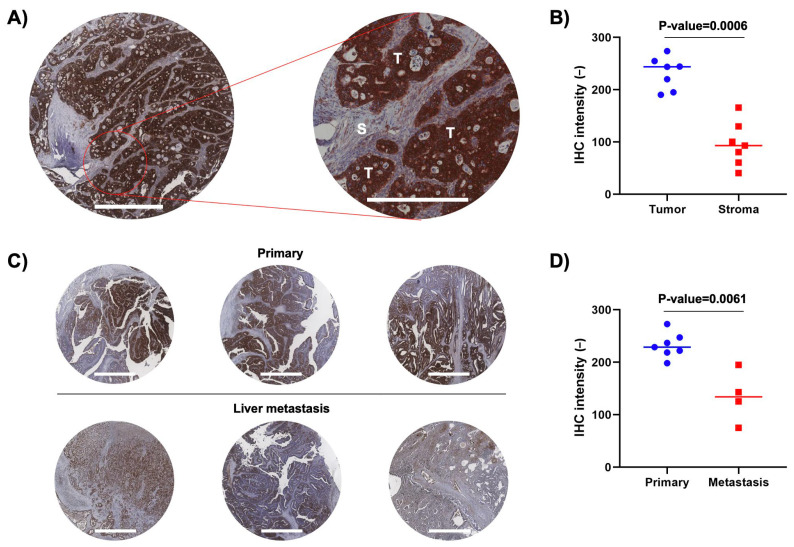
Immunohistochemistry (IHC) staining of TSPAN8 protein in primary and metastatic liver tissues of stage 4 CC. (**A**) A representative image from IHC staining of TSPAN8 protein in stage 4 primary CC tissues (left, scale bar: 1000 µm) and its enlarged image (right, scale bar: 500 µm). “T” shows the tumor area, while “S” indicates the stroma area. (**B**) Quantified IHC intensity in tumor and stroma area of stage 4 primary CC tissues. (**C**) Representative images from IHC staining of TSPAN8 protein in primary and metastatic liver tissues of stage 4 CC. Scale bar, 1000 µm. (**D**) Quantified IHC intensity in primary and metastatic liver tissues of stage 4 CC. The *p*-value was calculated using the Mann–Whitney U-test.

To validate a certain part of our finding, IHC staining of TSPAN8 protein in primary and metastatic liver tissues of stage 4 CC was performed. TSPAN8 protein was selected because TSPAN8 gene showed the highest expression among the differentially expressed genes in the tumor compartment of stage 4 primary CC tissues. Figure 7A presents a representative IHC image of TSPAN8 staining in stage 4 primary CC tissues. The expression of TSPAN8 protein was significantly overexpressed in the tumor compartment (T) in comparison to the stroma region (S), which includes the immune and fibroblast compartments (Figure 7A,B). Furthermore, representative images from IHC staining of TSPAN8 protein in primary and metastatic liver tissues of stage 4 CC are shown in Figure 7C. The reduction in TSPAN8 expression was significantly observed in metastatic liver tissues compared to primary CC tumor tissues (Figure 7D), which is well consistent with the above transcriptomic analysis. These results not only support a certain part of our spatial transcriptomic analysis in primary and metastatic liver tissues of stage 4 CC but also suggest TSPAN8 as a biomarker or target for tumor-specific regions.

## 4. Discussion

The spatial transcriptomic analysis of tetraspanins in stage 4 CC tissues provides valuable insights into their spatial expression patterns, differential expression profiles, and potential clinical implications. This discussion section highlights the key findings of the study and contextualizes them within the existing literature, discussing their implications for CC biology and their potential as diagnostic markers and therapeutic targets.

The spatial transcriptomic profile of tetraspanins in stage 4 primary CC tissues offers a comprehensive view of these cell surface proteins within the tumor microenvironment. This study involved spatial transcriptomic profiling of 33 human tetraspanins in 36 AOI within stage 4 primary CC tissues. Segmentation into three distinct compartments (CD45+ for immune cells, SMA+ for fibroblasts, and PanCK+ for tumor cells) enabled the identification of cell populations within the tissue samples. The cell deconvolution results successfully confirmed the presence and composition of different cell types within their respective compartments. Colorectal adenocarcinoma was identified as the primary population in the tumor compartment, while immune cells, such as macrophages, mast cells, and CD4+ alpha beta T cells, were prevalent in the immune compartment. The fibroblast compartment consisted mainly of fibroblasts. Spatial transcriptomic analysis revealed different expression patterns of tetraspanins within different tumor regions of primary tissues of stage 4 CC. Tetraspanin genes exhibited spatial heterogeneity with specific overexpression in different sub-tumor regions, indicating their potential roles in various aspects of CC progression. In the tumor compartment, several tetraspanins (CD9, CD151, TSPAN1, TSPAN3, TSPAN8, and TSPAN13) were specifically overexpressed, suggesting their involvement in tumor growth and proliferation in stage 4 primary CC. This result is consistent with their reported roles in promoting cancer progression [11,12,21,22,23,24]. The fibroblast compartment showed significant overexpression of TSPAN9, indicating its potential role in tumor invasion and metastasis in stage 4 primary CC. This is consistent with the reported roles of TSPAN9 in the promotion of cancer cell motility, invasion, and metastasis in osteosarcoma [25]. In the immune compartment, CD53 was specifically overexpressed, indicating its potential role in the immune modulation of stage 4 primary CC. Consistent with the present result, earlier studies have demonstrated the immune role of CD53 in CC [7,26]. The spatial heterogeneity observed for tetraspanins highlights their various roles in different aspects of CC progression, including tumor proliferation, invasion, and interactions with the tumor microenvironment in primary tumor. This observation also aligns with previous studies, which demonstrated the heterogeneity of CC tumors, and supports the notion that different tumor compartments contribute to tumor growth, invasion, and metastasis [27]. The identified overexpressed tetraspanins have the potential to serve as promising biomarkers and therapeutic targets specific to particular sub-tumor regions in stage 4 primary CC. For instance, CD151 and TSPAN8, which have been proposed as potential therapeutic targets for CC [10,28], were overexpressed in the tumor region of stage 4 primary CC tumors. Targeting CD151 and TSPAN8 in this context may help develop region-specific therapies for inhibiting cancer cell growth in stage 4 primary CC tumors. CD151 and TSPAN8 could also be identified as biomarkers for tumor cells in the primary tumor. Furthermore, overexpression of TSPNA9 related to tumor invasion may be used as a therapeutic target to reduce primary tumor invasion [25]. CD53 was overexpressed in the immune infiltrated areas of stage 4 primary CC tumors. Therefore, the targeting of CD53 could be explored to modulate immune responses and improve the effectiveness of immunotherapy for the immune microenvironment of stage 4 primary CC tumors [29]. These findings emphasize the complexity of CC and underscore the importance of a spatially resolved approach to gain a comprehensive understanding of the molecular landscape within the tumor microenvironment. The spatial transcriptomic analysis of tetraspanins in stage 4 primary CC tissues provides additional insights into the intricate interplay of these proteins in different tumor regions. These findings have implications for precision medicine and the development of targeted therapies, which can address the specific molecular characteristics of various sub-tumor regions in stage 4 primary CC. 

Moreover, differential gene expression analysis revealed significant alterations in tetraspanin expression levels in stage 4 primary CC tumor compared to patient-matched metastatic liver lesion. Cell deconvolution analysis provided important information on the cellular composition of both primary and metastatic tumors. The analysis confirmed that colorectal adenocarcinoma cells remained the predominant population in the tumor compartment of metastatic liver tumor. This confirms the presence of CC cells in the metastatic site, validating the nature of secondary tumor. Fibroblasts were abundantly present in the fibroblast compartment of both primary CC and metastatic liver tissues. Notably, the analysis revealed significant changes in the immune compartment between primary and metastatic tumors. There was an increase in the population of CD4+ alpha beta T cells in the immune compartment of metastatic tumor compared to the primary tumor. This increase in CD4 + alpha-b beta T cells supports previous research and may have implications for the immune response within the metastatic microenvironment [30,31]. Conversely, the population of M1 macrophage cells decreased in the immune compartment of metastatic tumor compared to the primary tumor. This shift toward M1 macrophages, which are often associated with pro-inflammatory and anti-tumor responses, may indicate changes in the immune milieu [32,33]. Collectively, these observations suggest that the most significant alterations in cell sub-populations occurred in the immune compartment of metastatic tumor compared to the primary tumor. This highlights the dynamic changes in the immune landscape as CC progresses to liver metastasis. Understanding these cellular changes is crucial to unraveling the complex interactions within the tumor microenvironment during metastasis. It may also inform the strategies for targeting specific cell populations or modulating the immune response to improve treatment outcomes in metastatic CC.

When comparing stage 4 primary CC tumor with patient-matched metastatic liver lesion, differential gene expression analysis showed notable changes in tetraspanin expression levels in sub-tumor regions. In particular, the alteration in tetraspanins is likely influenced by specificity of the organ site because different organs provide unique microenvironments, which can support or hinder metastatic growth [34]. In the tumor compartment of the metastatic site, the expression patterns of several tetraspanin genes, including CD63, CD82, CD151, TSPAN3, TSPAN4, TSPAN12, TSPAN13, TSPAN15, TSPAN31, TSPAN32, and UPK1B, were significantly upregulated. These upregulated tetraspanins in tumor cells within metastatic tumor are likely associated with several key factors. The increased expression of these tetraspanins suggests their involvement in enhancing the metastatic potential of cancer cells. They may facilitate processes such as invasion, intravasation into blood vessels, circulation in the bloodstream, and extravasation at the metastatic site. Since the liver is a common site of metastasis in CC, these upregulated tetraspanins may play specific roles in liver metastasis, including interactions with liver-resident cells or adaptations to the physiological conditions of the liver. These tetraspanins may contribute to the adaptation of tumor cells to the liver microenvironment, making them particularly relevant in liver-specific metastatic processes. This also indicates their potential as oncogenic drivers in stage 4 CC. These findings are consistent with many previous studies implicating these tetraspanins in cancer progression and/or metastasis [7,12,35,36,37,38]. Conversely, CD9, TSPAN6, and TSPAN8 were downregulated in the tumor compartment of metastatic tumor. Downregulated tetraspanins could influence the behavior of tumor cells within the liver microenvironment. This might involve reduced interactions with specific cell types or altered signaling pathways, which affect tumor cell behavior. This may also suggest their potential tumor-suppressor functions in stage 4 primary CC. These results are consistent with earlier research suggesting that these tetraspanins influenced tumor cell behavior in the tumor microenvironment [7,11,39]. Therefore, differential regulation of tetraspanin genes in the tumor compartment of the metastatic site suggests their involvement in organ-specific metastatic processes and modulation of tumor cell behavior within the liver microenvironment. The distinct tetraspanin expression patterns between stage 4 primary CC tumors and metastatic liver lesions offer potential clinical benefits. Targeting upregulated tetraspanins, such as CD63, CD82, and CD151, presents a novel strategy for hindering metastatic growth by impeding invasion and intravasation. These upregulated tetraspanins may serve as diagnostic markers for liver-specific metastasis in stage 4 CC, guiding tailored treatments. The differential regulation of tetraspanins, especially the downregulation of CD9, TSPAN6, and TSPAN8 in the metastatic site, provides prognostic insights into altered interactions within the liver microenvironment. Monitoring these tetraspanins offers valuable information on metastatic progression. Additionally, downregulated tetraspanins may serve as therapeutic targets with tumor-suppressor functions, indicating the strategies for intervention. These findings suggest nuanced approaches for treating liver-specific metastasis, pending further research and clinical validation. 

The upregulation of TSPAN13, TSPAN31, and UPK1B genes in the fibroblast compartment of metastatic tumor suggests their potential significance in the context of tumor–fibroblast interactions, metastasis, and the distinct characteristics of the metastatic microenvironment. These elevated tetraspanins might result in promoting cancer cell invasion, facilitating extracellular matrix remodeling, or aiding in the formation of a pro-metastatic microenvironment, which supports cancer cell survival and proliferation [40,41,42]. The unique features of the metastatic niche, which differ from the primary tumor microenvironment, may be influenced by these upregulated tetraspanins in fibroblasts [43]. These changes could play a role in the adaptation of fibroblasts to the specific conditions of liver metastasis. Clinical applications may include interventions aiming to modulate these upregulated tetraspanins, potentially offering new avenues for therapies, which specifically target the fibroblast compartment in liver metastasis. Further research is essential to unravel the precise mechanisms and validate these findings for practical clinical use.

Significant gene upregulation of these tetraspanins (CD53, ROM1, TSPAN3, TSPAN12, TSPAN15, and UPK1B) within immune cells in metastatic tumor suggests their potential involvement in the influence of immune responses. These upregulated tetraspanins likely contribute to enhanced immune cell activation, potentially improving the immune system’s ability to recognize and respond to tumor cells in the liver metastatic microenvironment [29,44,45]. The downregulation of CD63 and TSPAN32 in immune cells within metastatic tumor suggests a potential reduction in immune cell activation and responsiveness [26,44,45]. This downregulation might compromise the immune system’s ability to mount effective anti-tumor responses in the metastatic setting. It is also important to consider that these changes in tetraspanin gene expression should be related to variations in immune cell populations between primary and metastatic tumors, as mentioned earlier. These findings provide insights into the distribution of tetraspanin-expressing immune cells within liver metastases, which can be distinct from the immune compartment of primary tumor. Recognizing these alterations in tetraspanin gene expression is vital for tailoring immunotherapeutic strategies to optimize responses specifically against liver metastases in stage 4 CC patients.

In addition to the spatial transcriptomic analysis, we validated the expression of TSPAN8 protein through IHC staining in primary and metastatic liver tissues of stage 4 CC. TSPAN8—chosen for its highest expression among differentially expressed genes in the tumor compartment of stage 4 primary CC tissues—exhibited significant overexpression in the tumor compartment in comparison to the stroma region, including immune and fibroblast compartments. We also observed a significant reduction in TSPAN8 expression in metastatic liver tissues compared to primary CC tissues, aligning with the transcriptomic analysis. These results not only support our spatial transcriptomic exploration but also emphasize TSPAN8 as a potential biomarker or target for the tumor-specific region. Validation of the other tetraspanins should be performed in the future.

Numerous studies have explored spatial transcriptomics in various cancers, including colorectal cancer [46,47]. However, to the best of our knowledge, a study specifically focusing on the spatial transcriptomic landscape of tetraspanins in both primary and metastatic liver tissues of stage 4 CC has not been reported. While this study offers valuable information on the spatial transcriptomic landscape of tetraspanins in stage 4 CC tissues, it is essential to acknowledge some limitations and potential contradictory/unexpected findings for a nuanced interpretation. The sample size was relatively small, and the study was retrospective, possibly introducing biases. The findings need validation in larger cohorts and prospective studies to establish robustness and generalizability. Potential discrepancies in the differential expression of certain tetraspanins in primary and metastatic liver tissues of stage 4 CC may arise in the future. Considering cellular heterogeneity within the tumor microenvironment is crucial, as unexpected variations in tetraspanin expression in other cell types or compartments may indicate complex interactions, which are not fully understood. Challenges persist in reconciling transcriptomic and protein-level analyses. Additionally, further validations and functional experiments, such as in vitro and in vivo studies, are necessary to elucidate the expression and specific roles of tetraspanins in stage 4 CC progression and metastasis.

Collectively, the spatial transcriptomic analysis of tetraspanins in stage 4 CC tissues unraveled their spatially resolved distinct expression patterns and differential expression profiles. These findings provide important insights into the spatial organization and potential functional roles of tetraspanins in CC biology. The identification of specific tetraspanins as potential diagnostic markers and therapeutic targets holds promise for improving patient outcomes in stage 4 CC. Further research is warranted to unravel the underlying mechanisms and validate the clinical utility of tetraspanins for stage 4 CC.

## 5. Conclusions

Our study represents the first analysis of the spatial transcriptomic landscape of tetraspanins in stage 4 CC tissues. The findings uncover distinct spatial expression patterns in both primary CC and metastatic liver tissues, emphasizing the relevance of specific tetraspanins in the progression and liver metastasis of stage 4 CC. These results significantly contribute to our understanding of CC biology, suggesting the potential of particular tetraspanins as valuable diagnostic markers and promising therapeutic targets in stage 4 CC. Further research is imperative to elucidate their precise roles, and subsequent efforts should prioritize the development of targeted therapies tailored to specific tetraspanin profiles, followed by rigorous clinical validation. 

## Data Availability

GeoMx DSP data generated from this study are available in the Supplementary File S2. Other data are also available from the corresponding author upon request.

## Supplementary Materials

The following supporting information can be downloaded at: https://www.mdpi.com/article/10.3390/life14010126/s1, Supplementary File S1: Scheme S1, Figures S1–S4, and Tables S1 and S2; Supplementary File S2: GeoMx DSP data.
