# Supplementary material for: Spatial Transcriptomic Profiling of Tetraspanins in Stage 4 Colon Cancer from Primary Tumor and Liver Metastasis
Source: Life (Basel). 2024 Jan 15;14(1):126. doi: 10.3390/life14010126 (PMC10817616; doi:10.3390/life14010126)
Supplement: Supplementary file 1 [file life-14-00126-s001.zip › Supplementary File S1.pdf]

Supplementary File S1

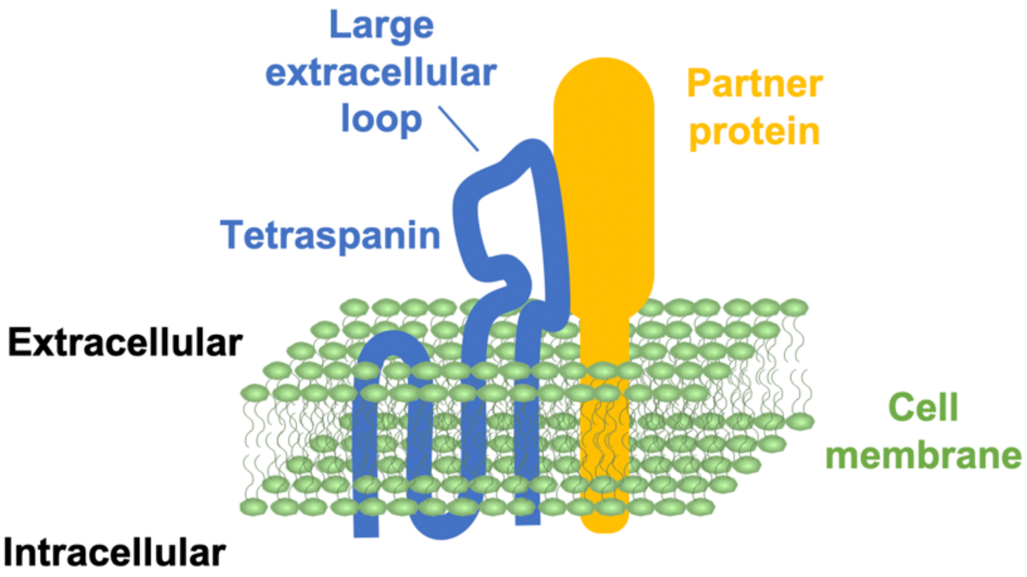

Scheme S1 Tetraspanins' structure and their molecular association.

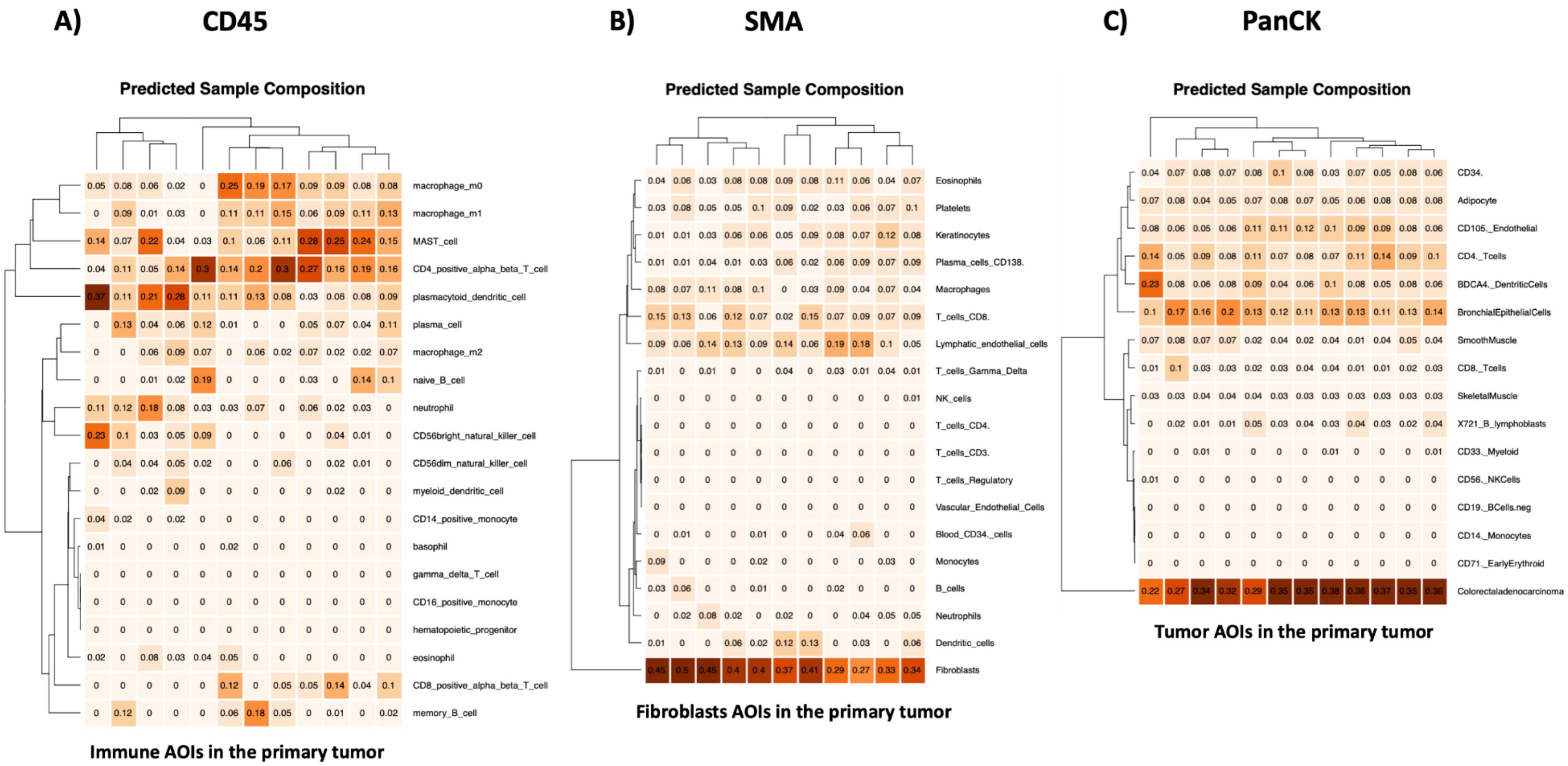

Figure S1 Heatmaps showing sample composition predicted by the Gene Expression Deconvolution Interactive Tool (GEDIT, https://webtools.mcdb.ucla.edu/) within (a) immune, (B) fibroblast, and (C) tumor areas of interest (AOI) in stage 4 primary colon cancer (CC) tissues. The number in the heatmaps represents the fraction of mRNA originating from each cell type, which is an effective measure of the transcriptional contribution of each cell type in each AOI.

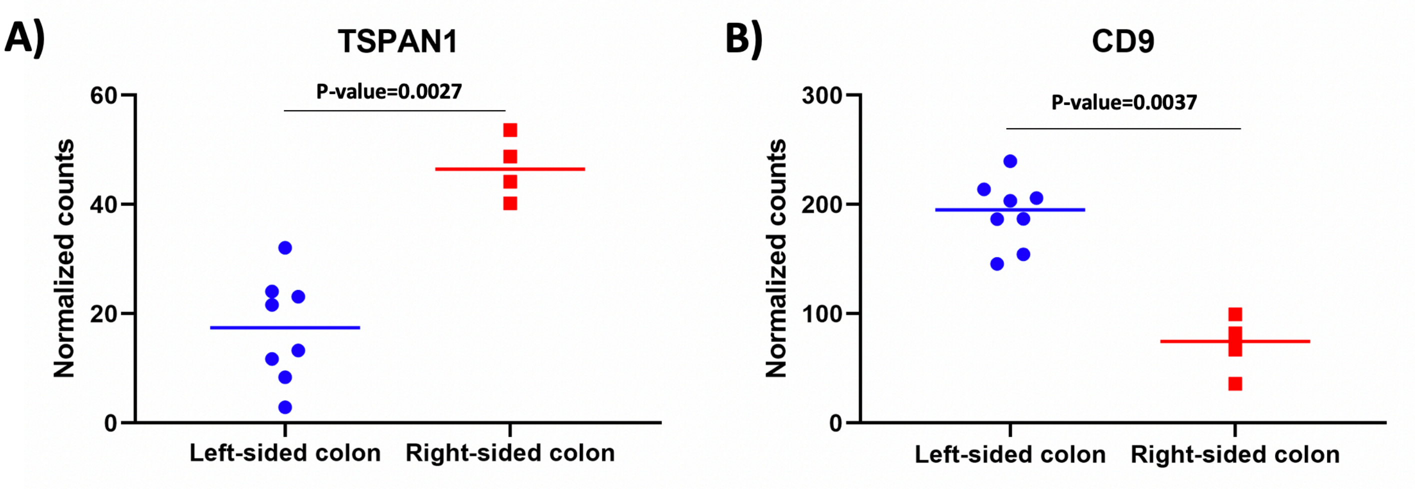

Figure S2 The plots depict the differential gene expression levels of (A) TSPAN1 and (B) CD9 in stage 4 primary colon cancer (CC) tissues from left-sided and right-sided colons. The P-value was calculated by T Test.

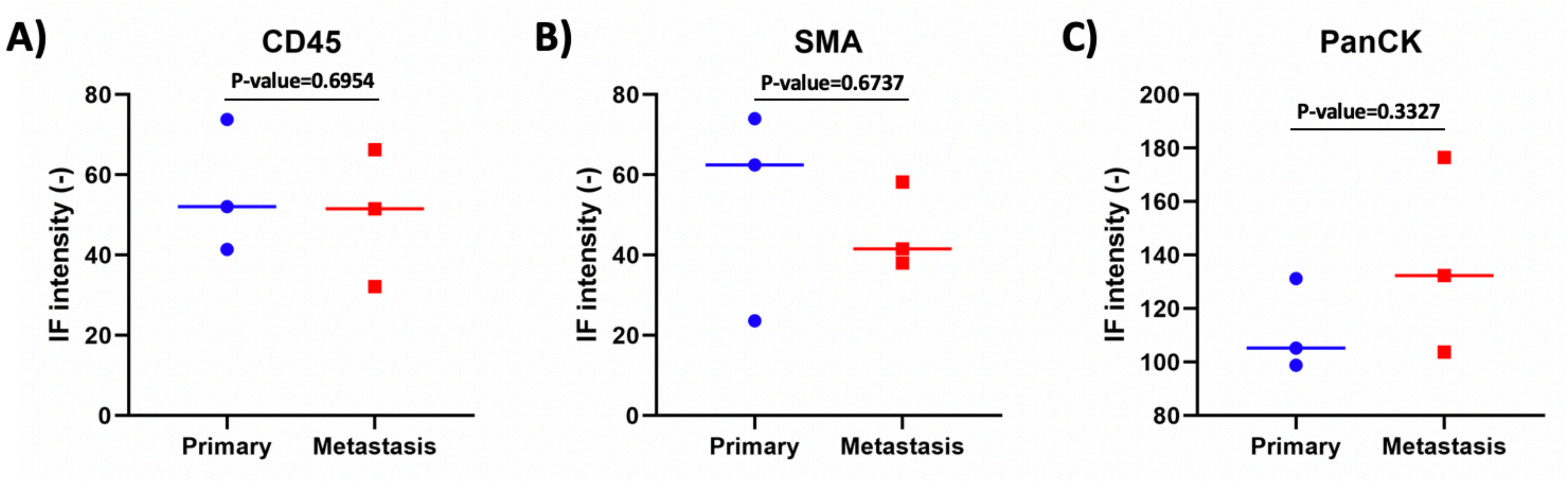

Figure S3 Quantified immunofluorescence (IF) intensity of (A) CD45, (B) SMA and (C) PanCK in IF images of primary and metastatic liver tissues of stage 4 colon cancer. IF intensity was quantified using ImageJ program. The P-value was calculated by T Test or Mann-Whitney U test.

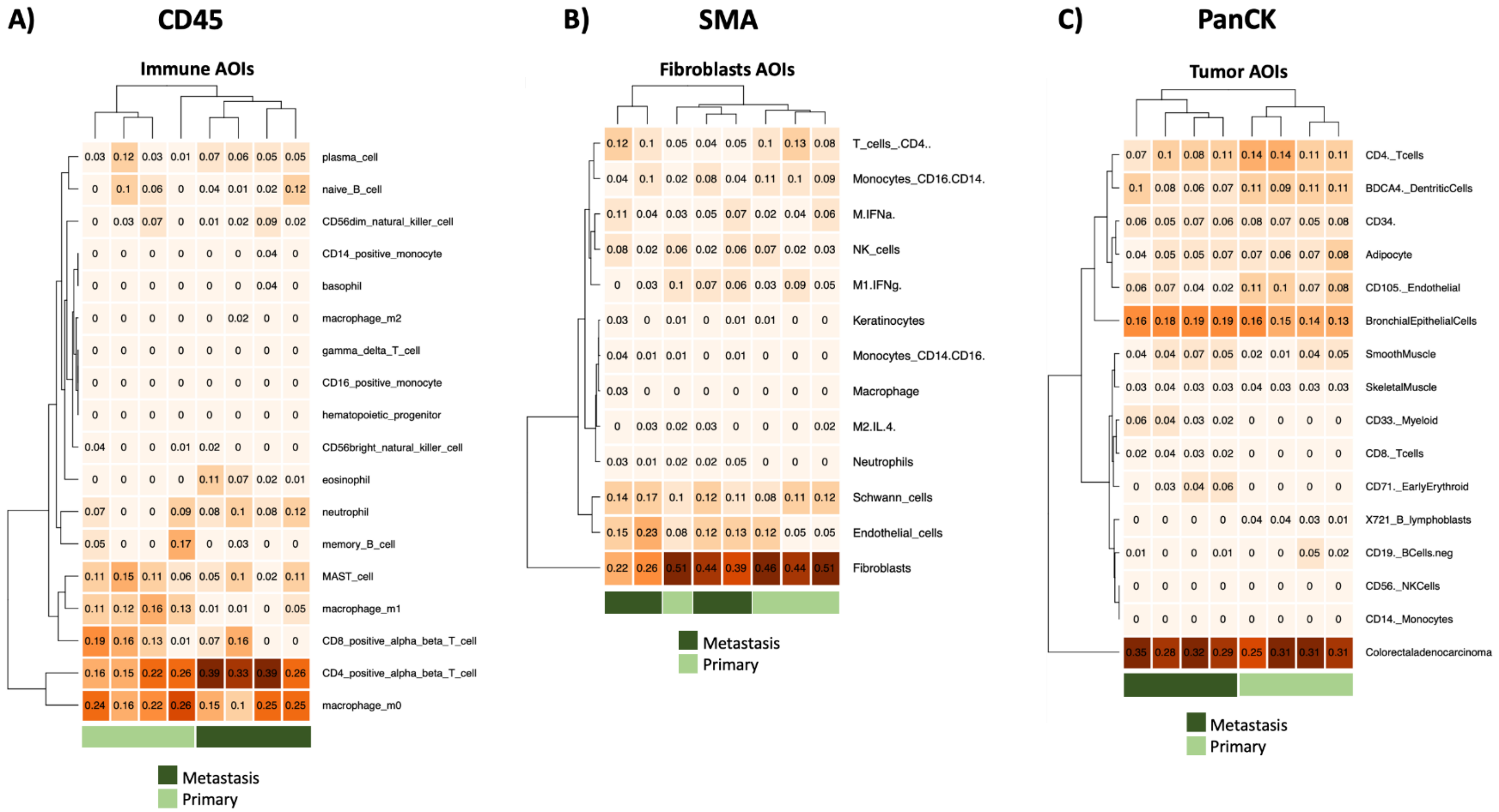

Figure S4 Heatmaps showing sample composition predicted by the Gene Expression Deconvolution Tool (GEDIT, https://webtools.mcdb.ucla.edu/) within (a) immune, (B) fibroblast and (C) tumor areas of interest (AOI) in stage 4 primary colon cancer (CC) and matched metastatic liver tissues. The number in the heatmaps represents the fraction of mRNA originating from each cell type, which is an effective measure of the transcriptional contribution of each cell type in each AOI.

Table S1 Statistical analysis result for spatial transcriptomic profiling of tetraspanins in stage 4 primary CC tissues

Marker_gr	CD45		SMA		PanCK		Kruskal-Wallis H test
Gene	Mean	Std. Deviation	Mean	Std. Deviation	Mean	Std. Deviation	P-value
TSPAN1	6.0806	4.09149	6.2045	4.132	26.9902	16.73154	0.001
TSPAN2	4.9673	2.888	7.6842	4.01088	2.9004	2.09333	0.001
TSPAN3	7.6781	2.70154	10.4815	6.55814	18.8105	6.46466	<0.001
TSPAN4	4.1269	2.253	6.9776	4.96096	1.7174	1.07163	<0.001
TSPAN5	3.6338	1.82518	5.3019	2.56837	4.5991	2.33546	0.23
TSPAN6	4.4663	2.10865	5.2611	3.42591	9.5758	2.78301	0.001
TSPAN7	2.908	2.22451	3.8346	2.06956	2.5461	1.03125	0.222
TSPAN8	23.3515	16.12737	12.9596	8.35471	142.1077	49.14467	<0.001
TSPAN9	5.8052	1.79484	13.939	7.73993	3.588	1.16147	<0.001
TSPAN10	3.0479	1.46784	3.6448	1.16377	2.0514	1.17873	0.01
TSPAN11	4.8059	2.15159	4.7398	2.13837	5.0429	2.16573	0.952
TSPAN12	1.9942	1.30958	3.9418	2.08759	1.2203	1.09729	<0.001
TSPAN13	9.7445	5.07469	6.5494	4.56533	18.151	5.00148	<0.001
TSPAN14	9.8476	4.13854	7.5389	5.57007	8.5047	2.30002	0.285
TSPAN15	6.8887	2.74992	7.0574	4.77082	12.5031	3.17113	0.001
TSPAN16	2.5772	1.53028	3.4338	2.05678	1.5542	1.15946	0.012
TSPAN17	7.1087	4.40528	6.3597	3.40753	8.4435	1.70482	0.102
TSPAN18	4.1207	2.37507	6.8712	3.65913	2.8604	3.44466	0.002
TSPAN19	3.5383	1.8612	3.4028	1.33293	2.0441	1.15853	0.031
UPK1B	2.7907	1.5806	4.3275	3.05514	1.3165	1.05237	<0.001
UPK1A	2.9592	2.08471	3.8771	1.2397	1.5409	0.96764	0.001
PRPH2	4.9163	4.12568	4.2903	1.84968	2.3552	1.07085	0.043
ROM1	3.03	1.8691	5.4163	2.37827	1.7281	1.38687	0.001
CD151	14.1528	7.41534	22.2123	8.94673	34.7515	6.34395	<0.001
CD53	21.1965	6.6446	6.4864	4.27744	3.1728	2.21646	<0.001
CD37	6.6785	3.30447	4.6467	2.17572	1.5484	1.12446	<0.001
CD82	12.6678	3.59809	11.4213	5.88186	10.3606	3.87634	0.423
CD81	27.0914	12.44797	26.0418	10.06448	25.0176	5.85978	0.622
CD9	42.5826	28.30439	29.5479	16.09301	151.6746	65.96202	<0.001
CD63	81.3374	38.33011	105.7647	29.94588	88.3136	33.57356	0.183
TSPAN31	4.0201	1.4467	3.7317	1.909	3.2397	0.88007	0.675
TSPAN32	6.1651	3.71207	4.4791	1.96779	2.6297	2.06999	0.006
TSPAN33	5.1889	3.21303	3.3212	1.18717	1.9603	1.13323	0.002

Table S2 Statistical analysis result for spatially resolved differential gene expression levels of tetraspanins in primary and metastatic liver tissues of stage 4 CC

Marker	CD45				Mann-Whitney U test	Marker	SMA				Mann-Whitney U test	Marker	PanCK				Mann-Whitney U test
Site_gr	Primary		Metastasis			Site_gr	Primary		Metastasis			Site_gr	Primary		Metastasis		
Gene	Mean	SD	Mean	SD	P-value	Gene	Mean	SD	Mean	SD	P-value	Gene	Mean	SD	Mean	SD	P-value
TSPAN1	6.8382	2.81771	5.5857	3.93807	0.561	TSPAN1	4.3941	1.84243	5.9049	3.73592	0.661	TSPAN1	20.1283	5.69165	28.978	13.78788	0.149
TSPAN2	3.9704	1.60619	5.6974	3.60034	0.386	TSPAN2	9.1419	4.31589	9.1581	6.23012	1	TSPAN2	2.3601	0.61631	1.9443	0.18587	0.386
TSPAN3	8.6092	2.16619	16.8687	5.56179	0.02	TSPAN3	9.5329	2.79457	18.0031	7.57694	0.11	TSPAN3	16.6564	1.6835	56.3589	7.21306	0.021
TSPAN4	6.5935	1.29218	6.4794	5.11448	1	TSPAN4	7.3359	5.83435	9.5693	3.41942	0.386	TSPAN4	1.4662	0.90818	3.2347	0.76708	0.043
TSPAN5	4.0855	1.71955	3.3514	1.48205	0.306	TSPAN5	5.2878	2.98275	6.4143	2.09578	0.468	TSPAN5	3.0628	0.61917	2.3018	0.23663	0.149
TSPAN6	3.9259	1.18624	2.5694	1.28349	0.146	TSPAN6	3.9844	3.33814	4.1187	3.21929	0.77	TSPAN6	9.0025	2.99555	4.8869	0.98077	0.043
TSPAN7	1.4121	1.07252	3.128	1.54795	0.08	TSPAN7	2.8115	1.86673	3.7995	3.47902	0.773	TSPAN7	1.9651	0.88754	1.7489	0.48948	0.564
TSPAN8	28.9372	14.26428	10.3894	4.01962	0.083	TSPAN8	11.9347	4.16015	13.154	3.77141	0.468	TSPAN8	127.2074	40.97045	66.5034	14.63653	0.021
TSPAN9	6.8402	1.08111	4.8037	2.04367	0.149	TSPAN9	17.4273	9.38748	8.8573	6.61548	0.144	TSPAN9	3.3885	0.39295	2.8248	0.53891	0.248
TSPAN10	2.4935	1.20073	2.4577	0.77397	0.77	TSPAN10	2.9232	0.79605	3.2839	3.82232	0.386	TSPAN10	1.7292	1.17202	1.6145	0.40204	0.564
TSPAN11	5.2297	1.91254	5.8091	3.3039	1	TSPAN11	5.2133	2.68112	3.7749	3.47723	0.191	TSPAN11	4.5812	2.02445	2.8362	0.56435	0.083
TSPAN12	1.0668	0.43792	2.9046	1.65195	0.02	TSPAN12	2.197	0.56228	4.1432	3.40795	0.465	TSPAN12	0.6541	0.11551	1.2913	0.16508	0.021
TSPAN13	8.1681	0.66906	8.1551	4.26076	0.248	TSPAN13	3.2583	1.75018	17.5366	2.76113	0.021	TSPAN13	15.0886	2.48867	37.3151	8.45963	0.021
TSPAN14	10.886	3.28844	9.4956	3.16762	0.386	TSPAN14	9.5143	7.37582	7.0281	1.73778	0.773	TSPAN14	8.2303	1.46541	7.2132	0.78101	0.248
TSPAN15	7.0172	1.38555	11.7299	4.2294	0.02	TSPAN15	5.5857	2.35041	8.9555	2.47897	0.059	TSPAN15	12.2895	4.23642	20.0127	2.50717	0.021
TSPAN16	1.771	0.84125	2.0108	0.25799	1	TSPAN16	1.9177	0.77487	4.757	3.05441	0.081	TSPAN16	1.1483	0.40727	1.2013	0.45091	1
TSPAN17	6.5026	1.97267	5.0271	2.90455	0.386	TSPAN17	7.8386	4.39233	8.1207	4.24567	1	TSPAN17	8.9303	1.57349	8.2284	1.14732	0.248
TSPAN18	2.33	1.49671	3.0163	1.23054	0.468	TSPAN18	5.5857	3.12798	5.555	2.80474	0.885	TSPAN18	1.6712	0.84516	2.3904	0.28954	0.245
TSPAN19	2.4896	0.75734	4.1334	3.29128	0.663	TSPAN19	2.9418	2.02185	3.9222	3.58097	0.661	TSPAN19	2.3861	0.71638	1.5552	0.35927	0.149
UPK1B	2.1269	1.01332	13.0705	3.88489	0.021	UPK1B	3.4817	1.58731	25.2276	9.2474	0.021	UPK1B	1.174	0.48972	136.7947	21.68805	0.021
UPK1A	2.3735	1.24886	2.0108	0.25799	0.243	UPK1A	3.37	1.48625	4.6097	2.96372	0.386	UPK1A	1.2973	0.371	1.9024	0.3495	0.083
PRPH2	3.8621	2.37005	4.692	4.07103	1	PRPH2	3.9286	1.85181	3.6031	3.64818	0.564	PRPH2	1.914	1.08187	2.4329	0.26995	0.248
ROM1	1.6259	0.71213	4.3568	2.07599	0.043	ROM1	4.8223	3.16872	3.4558	3.67944	0.309	ROM1	1.2687	0.39289	1.3752	0.62523	0.386
CD151	20.1732	2.60669	16.0867	5.12098	0.248	CD151	29.9578	5.96747	26.6025	7.71789	0.564	CD151	39.6066	3.62329	56.9289	5.73383	0.021
CD53	24.7162	2.60458	35.1897	4.83174	0.021	CD53	6.4794	2.86126	7.4823	4.27388	0.767	CD53	2.0645	0.76654	3.07	0.45474	0.149
CD37	6.8934	2.38076	9.7191	5.06051	0.386	CD37	3.2583	2.70609	3.6031	3.64818	1	CD37	1.1383	0.43612	1.5195	0.41661	0.248
CD82	14.9454	2.60681	14.8579	3.52562	0.561	CD82	14.3366	6.38973	6.9667	3.55009	0.149	CD82	12.4447	2.5442	23.355	3.58921	0.021
CD81	33.5663	10.34854	36.1952	17.41393	1	CD81	29.3434	2.24912	54.9483	28.05389	0.191	CD81	25.5888	4.88306	20.764	3.07522	0.149
CD9	65.3887	20.46458	38.7646	22.82421	0.083	CD9	29.9951	12.46177	44.2066	6.82332	0.149	CD9	215.6143	16.53907	97.3731	11.28422	0.021
CD63	114.7438	9.67667	66.4695	17.74475	0.021	CD63	120.8926	15.0585	93.2071	29.97326	0.083	CD63	106.3512	4.51493	129.8636	2.45572	0.021
TSPAN31	3.4288	0.6075	3.0163	1.23054	0.564	TSPAN31	2.8115	1.16394	7.955	1.23187	0.019	TSPAN31	2.9386	0.49713	7.9058	1.8538	0.021
TSPAN32	6.5858	0.52022	3.91	1.33434	0.021	TSPAN32	4.4313	2.25035	5.1683	2.54821	0.564	TSPAN32	1.69	0.78012	2.8733	0.51638	0.021
TSPAN33	5.0692	1.03089	5.9208	2.40292	0.663	TSPAN33	2.197	0.56228	4.2905	3.13921	0.108	TSPAN33	1.8486	0.8006	1.9501	0.56787	0.561
